# Predictors of premarital screening and genetic counseling knowledge and attitude among deaf and hard hearing females in Tabuk, Saudi Arabia

**DOI:** 10.25122/jml-2021-0165

**Published:** 2022-03

**Authors:** Sahar Zedan Zaien, Amira Abdallah El-Houfey, Hanadi Alqahtani, Hanan Abd Elwahab El Sayed, Wafaa Taha Elgzar, Rasha Mohamed Essa, Hala Bayomy, Heba Abdel-Fatah Ibrahim

**Affiliations:** 1. Department of Special Education, College of Art & Education, Tabuk University, Tabuk, Saudi Arabia; 2. Community Health Nursing, Faculty of Nursing, Assiut University, Assiut, Egypt; 3. Department of Nursing, Jizan University, Jizan, Saudi Arabia; 4. Department of Medical Science Assistance, Community College, Tabuk University, Tabuk, Saudi Arabia; 5. Department of Community Health Nursing, Faculty of Nursing, Benha University, Benha, Egypt; 6. Department of Obstetrics and Gynecologic Nursing, Damanhour University, Damanhour, Egypt; 7. Department of Maternity and Childhood Nursing, Nursing College, Najran University, Najran, Saudi Arabia; 8. Department of Nutrition and Food Science, University of Tabuk, Tabuk, Saudi Arabia; 9. Department of Food Science and Technology, Damanhour University, Damanhour, Egypt; 10. Department of Obstetrics and Woman Health Nursing, Benha University, Benha, Egypt

**Keywords:** attitude, deafness, genetic counseling, knowledge, PMSGC – Premarital Screening and Genetic Counseling, KSA – Kingdom of Saudi Arabia

## Abstract

According to Saudi Arabia's 2030 vision, research should be directed to prevention, early detection, and intervention to reduce all types of disability. The current study aimed to investigate the predictors of Premarital Screening and Genetic Counseling (PMSGC) knowledge and attitude among deaf and hard hearing females' in Tabuk, Saudi Arabia. Descriptive correlational design was conducted on a convenience sample of 67 deaf and hard hearing students from the Tabuk region. Data were collected by an electronic questionnaire elaborated to the participants using sign language. The results revealed that most participants had incorrect answers regarding most PMSGC questions. Around two-thirds (68.7%, 65.7%, and 71.6%) of them strongly agree that PMSGC is very important, compatible with Islamic principles, and prevents family social and psychological problems, respectively. Older, urban area residents and university-educated participants have significantly higher knowledge and attitude scores than their peers (t=2.239, 4.887, 4.790 & p<0.05), respectively. Multiple regression shows that age (b=0.302, t=-2.795, p=0.007), education (b=0.336, t=2.425, p=0.019), mothers' education (b=0.314, t=2.345, p=0.023), and monthly income (b=-0.337, t=-2.503, p=0.015), are significant predictors of PMSGC knowledge. Furthermore, age (b=0.659, t=4.024, p=0.000), residence (b=0.293, t=2.233, p=0.030), education (b=-0.395, t=3.028, p=0.004), and type of disability (b=-0.443, t=-3.763, p=0.000) are significant predictors of PMSGC attitude. Although most deaf and hard hearing females have incorrect knowledge regarding PMSGC, most have a positive attitude. The study concluded that participants' education, mothers' education, and monthly income are significant predictors of PMSGC knowledge. Moreover, age, residence, education, and type of disability were significant predictors of higher PMSGC attitudes.

## INTRODUCTION

Early hearing loss significantly interferes with cognitive, physical, and psychosocial development. Around 15% of the adult population worldwide experienced some degree of hearing disability, and approximately one-third of them suffer from disabling hearing loss. The prevalence of disability is growing rapidly in the Kingdom of Saudi Arabia (KSA), as its prevalence is higher than previous World Health Organization (WHO) reports [1]. Among all Saudi regions, the Tabuk has the highest disability rate, with 4.3% of its population [2]. Consanguineous marriage is strongly associated with offspring hearing loss, and it has been common among Arab and some Muslim societies such as KSA for thousands of years.

According to WHO, around 466 million persons complain of moderate to severe bilateral hearing loss worldwide [3]. Hearing loss causes a significant burden on community institutions, especially the healthcare system. Chronic hearing loss necessitates long-life sophisticated medical care, costly rehabilitation, a unique educational system, and psychological care. The Premarital Screening and Genetic Counseling (PMSGC) program may be an effective intervention for preventing and early detection of hereditary hearing loss. Moreover, it provides voluntary genetic testing and premarital examination for future couples [4]. The PMSGC program is a part of primary prevention that enhances the wellbeing of women, their future family, and society. The PMSG program is a worldwide activity aiming to diagnose, treat unrecognized disorders, and reduce transmission of hereditary diseases to future offspring [5].

A recent study in Jeddah, KSA, indicated that genetic hearing loss is a prevalent problem among the Saudi population and can be prevented through PMSGC [6]. Furthermore, another study reported that one-half of their participants strongly agree that PMSGC should be mandatory by the governmental authority, and around one-third prefer obligatory marriage prevention among genetic disorder carriers [7]. Consequently, it is important to assess the predictors of PMSGC knowledge and attitude among general and deaf populations to build future health education programs based on scientific data.

Despite the importance of PMSGC for deaf and hard hearing populations, they are marginalized and neglected in such an important issue. Studies that explored deaf and hard hearing population knowledge and attitudes regarding PMSGC are scarce. Baldwin *et al.* investigated the effect of language and culturally appropriate pre genetic test counseling on the deaf and hard hearing adults' knowledge and attitude. They indicated that using sign language and tailoring culturally appropriate programs is very important in increasing deaf population awareness regarding PMSGC. They also emphasized the importance of PMSGC in controlling deaf and hard hearing occurrence in the offspring, reducing the discrimination experienced by this group, and improving their health and wellbeing [8].

According to the 2030 KSA vision, the prevention of disability should be a primary concern. Therefore, research should be directed toward prevention, early detection, and intervention to reduce disability [9]. Research to improve the deaf and hard hearing population's quality of life should be need-based as they are isolated from any health education programs because they can hardly communicate with their society. Furthermore, consanguineous marriage is widespread and deeply rooted in KSA culture. Consequently, this will increase the prevalence of many hereditary diseases, including hereditary deafness. PMSGC can significantly reduce this problem and its consequences for families and the community. Therefore, the first step in addressing the problem is exploring the predictors of premarital screening and genetic counseling knowledge and attitude among deaf and hard hearing females. Such data may pave the way for future evidence-based health education programs about PMSGC among deaf and hard hearing females.

## MATERIAL AND METHODS

We performed a descriptive correlational design study. The study was conducted at Tabuk University, Al Amal center for deaf females, and secondary schools (integrating hard-hearing students) in Tabuk city, Saudi Arabia.

### Participants and sampling

This study included a convenience sample of 67 participants. Given that this population represents a relatively small sector of the Tabuk population, the sample size formula is not applicable. However, all participants who corresponded with the inclusion criteria and agreed to participate in the study were included. The inclusion criteria represent unmarried Saudi females aged 15–45 years (childbearing period) who use and understand sign language and are free from mental or psychological problems and other genetic or health problems.

### Measuring tools and data collection

Data were collected using an electronic questionnaire. The questionnaire was elaborated to the participants using sign language by the principal investigator during data collection. The questionnaire was developed after reviewing the related literature and contains three parts. The first part includes basic data and personal/family history of the study participants, like age, residence, education, mother's education, consanguinity, types of disability, family history of deaf/hard hearing and genetic diseases, family history of PMSGC, and previous attendance of PMSGC educational programs. The second part contains the PMSGC quiz to assess the knowledge of deaf and hard hearing students. This part comprises seven questions; two are dichotomous questions, where the correct answer obtains "one", and the incorrect one gets "zero". For the other five multiple questions, the correct answer obtains "two"; incomplete "one", and incorrect or don't know scored "zero". The overall scores were obtained by summing the correct and incomplete answers, with a range of possible overall scores from 0 to 12, with higher scores corresponding to higher PMSGC knowledge levels. The quiz evaluates PMSGC definitions, hereditary and infectious blood diseases covered, benefits, the appropriate timing for the PMSGC, and validity of a healthy marriage certificate. The third part, the Likert scale, assesses deaf and hard hearing students' attitudes regarding PMSGC. It was tailored on a five-point Likert scale ranging from strongly disagree (1) to strongly agree (5). The overall attitude scores were obtained by summing the participants' responses, with a range of possible overall scores from 10 to 50, with higher scores corresponding to a more positive PMSGC attitude.

The instrument validity was tested by a jury of five experts in obstetrics and gynecology and a biostatistician. The instrument reliability was tested through Cronbach's alpha coefficient test. Cronbach's Alpha was 0.78, which showed an adequate internal consistency of the instrument.

Data were collected from September to November 2020 by the principal investigator (hearing disability specialist). The principal investigator contacted each of the previously mentioned settings to confirm approvals from the sitting administration and arrange for data collection. Because of the COVID-19 pandemic, data was collected through online classes using blackboard collaborate. A private online interview was conducted with 3–5 participants using sign language. The responsibility of a hearing disabilities specialist is to take the participants' informed consent at the beginning of online classes, distribute the questionnaire link among participants, elaborate questions using sign language, and ensure data completeness and accuracy.

### Data analysis

Data were analyzed via the Statistical Package for Social Science (SPSS, IBM, USA), version 23. The participants' basic data and personal/family history were represented using descriptive statistics. The participants' knowledge and attitude concerning PMSGC were described in terms of numbers, percentages, means, and standard deviations. Independent t-test and one-way ANOVA were performed to assess the associations between the participants' knowledge and attitude and their basic data, personal, and family history. The predictors of PMSGC knowledge and attitude were examined using multiple linear regression.

## RESULTS

### Demographic characteristics of study participants (N=67)

Around two-thirds, (65.7) of the participants are university students with a mean age of 23.64±4.04, and the majority (94%) are urban area residents. The participants' mother education is nearly equally distributed between illiterate (25.4%), read and write (22.4%), secondary education (25.4), and university education (26.9%). Two-thirds (61.2%) of the participants have a monthly income of 5,000 to 10,000 SAR per month, and 77.6% have consanguinity parents. Also, 59.7% of the participants are deaf, and 40% hardly hear. Furthermore, 61.2% of the participants have a family history of first-degree relatives of hereditary deafness or hard hearing, and 65.7% have no history of other genetic diseases. Furthermore, 95.5% of the participants did not attend any previous PMSGC programs, and 65.7% do not know if their parents received PMSGC or not.

Table 1 illustrates that most participants have incorrect answers regarding most PMSGC questions. A large proportion of the participants have incorrect answers to the questions about premarital screening definition (70.1%), genetic counseling definition (70.1%), hereditary blood diseases (76.1%), infectious blood diseases covered by PMSGC (77.6%), the appropriate time for PMSGC (70.1%), and validity of healthy marriage certificate (80.6%). Also, 55.2% of the participants had incorrect answers to the question related to PMSGC benefits.

**Table 1.** Participants' knowledge regarding PMSGC (N=67).

Participants' knowledge	Correct answer		Incomplete		Incorrect answer	
	N	%	N	%	N	%
**Definition of premarital screening**	6	9.0	14	20.9	47	70.1
**Definition of genetic counseling**	8	11.9	12	17.9	47	70.1
**Hereditary blood diseases covered by PMSGC**	9	13.4	7	10.4	51	76.1
**Infectious blood diseases covered by PMSGC**	13	19.4	2	3.0	52	77.6
**The appropriate time for the PMSGC**	20	29.9	-	-	47	70.1
**Validity of healthy marriage certificate**	13	19.4	-	-	54	80.6
**The benefits of PMSGC**	10	14.9	20	29.9	37	55.2

Table 2 demonstrates that most participants have a positive response for the majority of the attitude scale items. Around two-thirds (68.7%, 65.7%, and 71.6%) of the participants strongly agree that PMSGC is very important, compatible with Islamic law principles, and prevents social and psychological problems for the family. Also, 68.7%, 62.7%, and 65.7% of the participants support the mandatory PMSGC, considering it is indispensable if one of the spouses has hereditary deafness or hard hearing. Moreover, it allows the couple to decide the fate of their marriage.

**Table 2.** Participants' attitude towards PMSGC (N=67).

Attitude	Strongly disagree		Disagree		Indifference		Agree		Strongly agree	
	No	%	No	%	No	%	No	%	No	%
**PMSGC are very important**	0	0.0	0	0.0	10	14.9	11	16.4	46	68.7
**PMSGC can protect offspring from hereditary diseases and protect myself from infectious diseases**	2	3.0	4	6.0	8	11.9	13	19.4	40	59.7
**It scares me the thought that I might have children with genetic diseases**	0	0.0	2	3.0	18	26.9	13	19.4	34	50.7
**PMSGC are compatible with the principles of Islamic law**	2	3.0	2	3.0	10	14.9	9	13.4	44	65.7
**PMSGC prevent social and psychological problems of the family**	0	0.0	0	0.0	2	3.0	17	25.4	48	71.6
**PMSGC leads healthy and successful marriage**	0	0.0	0	0.0	8	11.9	19	28.4	40	59.7
**Consanguinity marriage leads to hereditary diseases such as deafness and hard hearing.**	0	0.0	8	11.9	10	14.9	19	28.4	30	44.8
**I support the mandatory PMSGC**	0	0.0	0	0.0	10	14.9	11	16.4	46	68.7
**PMSGC is very necessary if one of the spouses have hereditary deafness or hard hearing**	0	0.0	2	3.0	6	9.0	17	25.4	42	62.7
**PMSGC allow the couple to decide the fate of their marriage**	0	0.0	2	3.0	6	9.0	15	22.4	44	65.7

Table 3 shows that older participants (≥20 years) have significantly higher knowledge and attitude scores than their peers <20 years (t=2.239, p 0.029) and (t=4.887, p 0.000), respectively. Moreover, urban area residents have significantly higher attitude scores than rural areas (t=4.790, p 0.000). University students have significantly higher knowledge scores than secondary school students (t=4.462, p 0.000). In addition, the participants with a family history of PMSGC have significantly higher knowledge scores than their peers without a family history (t=18.705, p 0.000). Simultaneously, there are no significant associations between the participants' knowledge and attitude scores and mother's education, income, type of disability, and family history of deafness or hard hearing (p>0.05). Although the knowledge mean scores are higher among those who have attended previous PMSGC programs, the difference is not statistically significant (p>0.05).

**Table 3.** Association between participants' basic data and knowledge and attitude score regarding PMSGC (N=67).

Variable	Knowledge			Attitude		
	Mean±SD	t/F	P	Mean±SD	t/F	p
**Age**		2.239	0.029*		4.887	0.000**
<20 years	1.25±0.70			35.00±8.68		
≥20 years	3.93±3.35			45.32±5.11		
**Residence**		1.338	0.186		4.790	0.000**
Rural	1.50±0.57			31.42±0.58		
City	3.74±3.33			44.92±5.77		
**Education**		4.462	0.000**		1.193	0.237
Secondary School	1.43±0.66			42.78±8.41		
University	4.75±3.5			44.77±5.22		
**Mother education**		1.759	0.164		1.325	0.274
Illiterate	3.05±2.51			43.64±6.21		
Read and write	4.20±3.48			46.53±5.78		
Secondary education	2.47±3.28			44.47±8.07		
University education	4.72±3.51			42.11±5.41		
**Monthly income**		1.029	0.363		0.497	0.611
Less than 5,000 SR per month	4.42±3.17			45.28±4.93		
From 5,000 to 10,000 SR per month	3.63±3.20			43.46±6.58		
More than 10,000 SR per month	2.58±3.60			44.83±7.96		
**Type of handicaps**		0.566	0.574		0.702	0.485
Deafness	3.42±3.070			44.55±6.92		
Hard hearing	3.88±3.59			43.40±5.88		
**Family history of deafness or hard hearing**		2.945	0.091		0.460	0.500
1^st^ degree relatives	3.04±2.63			45.07±5.87		
2^nd^ degree relatives	3.84±3.62			41.07±8.21		
No history	5.15±4.35			44.00±6.08		
**Attendance of previous PMC program**		0.671	0.569		1.150	0.352
Yes	4.93±2.84			47.33±4.61		
No	3.29±0.41			44.12±6.57		
**Family history of premarital counseling**		18.705	0.000**		0.889	0.416
Yes	9.5000	2.07364		47.00±0.89		
No	4.8824	2.52196		45.05±4.80		
Don't know	2.6364	2.83738		43.59±7.37		

** P significant at ≤0.001; t – independent t-test; F – One way ANOVA.

Table 4 shows that age (®=0.302, t=-2.795, p=0.007), education (®=0.336, t=2.425, p=0.019), mothers' education (®=0.314, t=2.345, p=0.023), and monthly income (®=-0.337, t=-2.503, p=0.015), are significant predictors of PMSGC knowledge. The adjusted R^2^ shows that 0.423 of the participant knowledge can be predicted through the regression model of the related variables. Furthermore, age (®=0.659, t=4.024, p=0.000), residence (®=0.293, t=2.233, p=0.030), education (®=-0.395, t=3.028, p=0.004), and type of disability (®=-0.443, t=-3.763, p=0.000) are significant predictors of PMSGC attitude. The adjusted R^2^ shows that 0.489 can be predicted through the regression model of the related variables.

**Table 4.** Predictors of premarital counseling and genetic screening knowledge and attitude among the study participants (N=67).

Variables	Knowledge						Attitude					
	B	SE b	®	t	P	95.0% Confidence Interval for B	B	SE b	®	t	p	95.0% Confidence Interval for B
**Age**	1.268	0.454	0.302	2.795	0.007*	[0.359, 2.175]	13.135	3.264	0.659	4.024	0.000*	[6.598, 19.672]
**Residence**	-0.059	1.963	-0.004	-0.030	0.976	[-3.990, 3.872]	7.996	3.582	0.293	2.233	0.030*	[0.824, 15.168]
**Education**	2.357	0.972	0.336	2.425	0.019*	[0.410, 4.303]	-5.370-	1.773	-0.395	-3.028	0.004*	[-8.920, -1.819]
**Mother education**	0.921	0.393	0.314	2.345	0.023*	[0.134, 1.707]	-0.635-	0.716	-0.112	-.886-	0.379	[-2.069, 0.799]
**Monthly income**	-1.803	0.720	-0.337	-2.503	0.015*	[-3.245, -0.360]	0.642	1.314	0.062	.489	0.627	[-1.989, 3.27]
**Type of handicap**	1.331	0.851	0.196	1.563	0.124	[-0.373, 3.034]	-5.842-	1.553	-0.443	-3.763	0.000**	[-8.950, -2.732]
**Family history of deafness or hard hearing**	1.002	1.790	0.098	0.560	0.578	[-2.581, 4.585]	.729	.827	0.090	0.881	0.382	[-0.927, 2.385]
**Attendance of previous PMC program**	-1.963-	1.822	-.122	-1.078	0.286	[-5.611, 1.685]	-2.248-	3.324	-0.072-	-.677	0.501	[-8.903, 4.406]
**Family history of premarital counseling**	-0.375-	0.509	-0.097-	-0.737-	0.464	[-1.394, 0.644]	0.012	0.929	0.002	.013	0.990	[-1.847, 1.871]
**R^2^**				0.423						0.489		
**F(p)**				4.638 (0.000**)						6.065 (0.000)		

* P significant at ≤0.001; ** P highly significant at ≤0.001.

## DISCUSSION

The current study showed a positive family history of hereditary deafness or hard hearing in the first-degree relatives among nearly two-thirds of our study participants, and more than three-quarters of them had consanguinity parents. This result is in accordance with a study conducted in KSA by Warsy *et al.* [10], who found that nearly one-third of their participants' parents have consanguinity marriage from the first-degree cousins. They further added that consanguinity marriage among the participants was 37.9% [10]. In addition, Bener *et al.* revealed a high incidence of hereditary diseases in consanguineous marriage. The prevalence of hereditary deafness was found among more than one-quarter of the consanguineous current generation and their offspring [11]. In KSA, consanguinity marriage is popular and results in numerous genetic problems. In addition, deaf and hard hearing populations feel stigmatized and therefore prefer to marry their first-degree cousins, especially deaf ones, to avoid embarrassment and social isolation. This situation leads to a vicious circle that exaggerates the incidence and severity among offspring. Complete deaf and hard hearing extended families over more than three generations can be found in KSA [10].

The current study revealed that most of our study participants had a family history of hereditary deafness or hard hearing. However, most of them did not attend any previous PMSGC programs, which increases the magnitude of this problem for this neglected group. The shortfall of such educational programs among the deaf population may be attributed to decreased ability of health care providers to use sign language and identify their special needs.This leads to health inequalities and avoiding medical assistance by deaf individuals, especially for PMSGC. Therefore, health care professionals should be provided with training opportunities to interact effectively with deaf individuals through clinical experience [12, 13]. This point of view is supported by Godino *et al.*, who reported that most nurses did not have enough knowledge about genetic counseling as only a few of them studied medical genetics [14].

Most of the studied participants have incorrect answers regarding most questions of PMSGC. This finding is similar to Middleton *et al.*, who found that nearly half of sign language and hard hearing participants did not know about genetic counseling [15]. Moreover, Binshihon *et al.*, in their study in western Saudi Arabia, documented that nearly half of their participants, especially unmarried women, had poor knowledge about PMSGC. They further recommended early genetic screening and awareness programs for all the public schools and universities regarding PMSGC [16]. The deaf and hard hearing population's poor knowledge about PMSGC indicated the isolation and exclusion of this target group from the health services and educational programs.

Contrary to the current study findings, Moussa *et al.* reported that most Hail region students, KSA, had satisfactory knowledge about the national mandatory premarital screening program. However, they reported that their participants have insufficient knowledge regarding genetic and blood diseases covered by this program [17]. In Jordan, Altaany *et al.* found that more than three-quarters of young populations had sufficient knowledge about PMSGC [18]. Furthermore, Olwi *et al.* documented that undergraduate students enrolled in the study had good knowledge of genetics testing [19]. The differences between the current study results and other studies may be attributed to the study participants' nature. Until now, this is the first study that investigated the predictors of deaf and hard hearing females' knowledge and attitude towards PMSGC. Deaf and hard hearing populations suffered from isolation and inequalities in health education and services, although they urgently needed these services. Furthermore, 34% of the current study participants have secondary school education. The latter study group was conducted in normal, highly educated students; therefore, it is expected that they can easily reach health services and education.

Changing attitude toward PMSGC is the most important step in increasing the utilization of PMSGC. Surprisingly, most of our participants had a positive attitude regarding PMSGC. The previously mentioned study stated that more than half of their participants had a positive attitude toward PMSGC [16]. In addition, Moussa *et al.* documented that around three-quarters of the university students believed that it is important to perform PMSGC [17].

On the other hand, Middleton *et al.* reported that deaf and hard hearing adults had a negative attitude toward PMSGC, and more than half of them thought genetic testing could be more harmful than good [20]. The difference between the present study and Middleton *et al.* may be due to the different times the two studies were conducted. The latter study was conducted 22 years ago, where the culture, knowledge, and beliefs regarding PMSGC were utterly different. In addition, knowledge about hard hearing and defenses genetic roots was not evident at that time. Besides, PMSGC was not mandatory for 22 years; therefore, the population did not realize its importance.

The current study revealed that more than two-thirds of participants support the mandatory PMSGC, considering it is important and necessary if there is hereditary deafness or hard hearing. This finding is consistent with Middleton *et al.*, who found that more than half of deaf and hard hearing participants in their study think they should be screened and counseled for hereditary deafness to reduce its incidence in the next generation [15]. In addition, Altaany *et al.* found that most of their participants (93.9%) believed that genetic testing is a useful and important tool in diagnosing and forecasting genetic diseases [18]. Also, a study conducted by Al-Farsi *et al.* among adults at the South Batinah Governorate in Oman stated that the majority of their participants thought PMCGS was essential. In addition, about half of them reinforced that PMCGS should be compulsory and preferred to prevent consanguineous marriages to avoid developing hereditary diseases among offspring [21].

Furthermore, an important positive finding in our study is that around two-thirds of participants considered PMSGC compatible with Islamic law principles. According to the previous study conducted by Altaany *et al.*, 88.2% of their respondents thought genetic testing is not forbidden and permissible [18]. The benefit and welfare of the individuals and community are deeply rooted in Islamic rules. Islam advises future couples to disclose any hereditary or health problems before marriage and consider hiding such a problem as dishonesty.

This study showed that university students have significantly higher knowledge scores than secondary school students. The other two studies reported similar findings. First, Binshihon *et al.* stated that knowledge was higher among participants with high education (≥university degree) [16]. Second, Ibrahim *et al.* stated a positive relationship between the educational level and knowledge scores; as the educational level increases, the knowledge score increases [22]. These findings reflect the important role of education in confronting misconceptions and harmful traditions that may negatively affect the next generation's health and wellbeing. The benefit of education may be maximized by adding the topic of PMSGC in educational curricula.

Furthermore, an unexpected result was documented in our current study. No significant associations between the participants' knowledge, attitude, and family history of deafness or hard hearing (p>0.05). The presence of affected persons in the family with the same condition was supposed to increase awareness about the importance of PMSGC. Nevertheless, most deaf and hard hearing populations generally have a positive attitude toward PMSGC regardless of family history. The current study results agree with two Saudi studies that found no significant association between family history of PMSGC and their participant knowledge [16, 23].

In addition, our study illustrated that knowledge is higher among those who have attended previous PMSGC programs. These findings are similar to a Saudi study conducted by Olwi *et al.*, who reported that previous awareness of genetic testing through the educational curriculum was significantly associated with good genetics knowledge [19]. Also, Zaien *et al.*, who conducted an educational intervention to empower deaf and hard hearing females regarding PMSGC in Tabuk region KSA, concluded that educational intervention is an effective tool to increase deaf population knowledge and enhance their attitude [24].

It is worth mentioning that participants' age, education, mothers' education, and monthly income are significant predictors for PMSGC knowledge in this study. Similar findings were reported by Bener *et al.* They conducted multivariate regression analysis and found that the most common predictors for PMSGC knowledge were the participant's age, educational level, religion, household income, consanguinity, and occupational status [25]. In addition, Ibrahim *et al.* stated that education is the first and most important predictor for PMSGC knowledge, where university-educated girls had significantly higher knowledge than lower educated girls. In addition, monthly income is a positive predictor for high knowledge. They also highlighted the importance of extending the spectrum of genetic testing to include various genetic problems [22].

Furthermore, our study illustrated that participants' age and education are significant predictors of PMSGC attitude. The same result was reported by Olwi *et al.* [19]. PMSGC has become mandatory in KSA; this helps increase public awareness regarding its benefits for the family and community. In addition, it increased PMSGC acceptance among Saudi populations; therefore, a high positive attitude is expected from adults with higher education. Given the high incidence of congenital deafness and consanguineous marriage in KSA, the benefits of this study extend beyond the individual to the nation as a whole.

One limitation of this study is that it uses a convenience sample. In accordance, the study's findings cannot be generalized; however, deaf and hard hearing populations are a relatively small group and not suitable for randomization. In addition, the study did not include deaf and hard hearing male participants; therefore, further studies are needed to include the male population.

## CONCLUSIONS

Most deaf and hard hearing females have incorrect knowledge regarding PMSGC, although most have a positive attitude towards PMSGC. The vast majority support the mandatory PMSGC, considering it essential if one of the spouses has hereditary deafness or hard hearing. Participants' education, mothers' education, and monthly income are significant predictors of PMSGC knowledge. Furthermore, age, residence, education, and type of disability were identified as significant predictors of higher PMSGC attitudes. There are few studies about deaf and hard hearing population knowledge and attitude in Saudi Arabia and worldwide. This study offers significant data on the PMSGC knowledge and attitude of deaf and hard hearing females in Saudi Arabia. The availability of such data can enrich the database and direct future research to deeply investigate the barriers for deaf and hard hearing population adherence to PMSGC. This type of research can contribute to designing the most effective educational programs targeted to meet the needs and improve the quality of life for this neglected group in society. The deaf and hard hearing population is often isolated from health education programs and services.

## ACKNOWLEDGMENTS

### Conflict of interest

The authors declare no conflict of interest.

### Ethical approval

This study was approved by the Deanship of Scientific Research at the University of Tabuk (S-1441-0028, August 18, 2020). Formal approvals from data collection sittings were obtained via formal lines of authority by the principal investigator.

### Consent to participate

Informed consent was taken from each participant before data collection. All the information was confidential and was used only for research purposes.

### Funding

This study was funded by the Deanship of Scientific Research at the University of Tabuk Kingdom of Saudi Arabia.

### Personal thanks

The authors would like to express their Gratitude to the Deanship of Scientific Research at the University of Tabuk Kingdom of Saudi Arabia for their financial and technical support under code number (S-1441-0028).

### Authorship

SZZ collected and processed data. AAE wrote the original draft. HA contributed to data collection. HAEE developed the study concept and design. WTE performed the critical revision of the paper. RME supervised the study. HB conducted the literature review. HAI analysed data and draft manuscript preparation. All authors provided critical review to the study.

## References

[1] World Health Organization, World Health Organization (2012). WHO global estimates on prevalence of hearing loss. https://www.who.int/pbd/deafness/WHO_GE_HL.pdf.

[2] Bindawas SM, Vennu V (2018). The National and Regional Prevalence Rates of Disability, Type, of Disability and Severity in Saudi Arabia-Analysis of 2016 Demographic Survey Data. Int J Environ Res Public Health.

[3] World Health Organization, World Health Organization (2017). WHO Programme for Prevention of deafness and hearing loss. https://euracoustics.org/news/eaa-newsletter/2018/.

[4] Bozkurt G (2007). Results from the north cyprus thalassemia prevention program. Hemoglobin.

[5] Abd Al Azeem ST, Elsayed ET, El Sherbiny NA, Ahmed LA (2011). Promotion of knowledge and attitude towards premarital care: An interventional study among medical student in Fayoum University. J Public Health Epidem.

[6] Alshehri KA, Alqulayti WM, Yaghmoor BE, Alem H (2019). Public awareness of ear health and hearing loss in Jeddah, Saudi Arabia. S Afr J Commun Disord.

[7] Al Kindi R, Al Rujaibi S, Al Kendi M (2012). Knowledge and attitude of university students towards premarital screening program. Oman Med J.

[8] Baldwin EE, Boudreault P, Fox M, Sinsheimer JS, Palmer CG (2012). Effect of pre-test genetic counseling for deaf adults on knowledge of genetic testing. J Genet Couns.

[9] https://vision2030.gov.sa/en.

[10] Warsy AS, Al-Jaser MH, Albdass A, Al-Daihan S, Alanazi M (2014). Is consanguinity prevalence decreasing in Saudis?: A study in two generations. Afr Health Sci.

[11] Bener A, Mohammad RR (2017). Global distribution of consanguinity and their impact on complex diseases: Genetic disorders from an endogamous population, The Egyptian journal of medical human genetics.

[12] Hoang L, LaHousse SF, Nakaji MC, Sadler GR (2011). Assessing deaf cultural competency of physicians and medical students. J Cancer Educ.

[13] Adib-Hajbaghery M, Rezaei-Shahsavarloo Z (2015). Nursing students' knowledge of and performance in communicating with patients with hearing impairment. Jpn J Nurs Sci.

[14] Godino L, Turchetti D, Skirton H (2013). Genetic counseling: a survey to explore knowledge and attitudes of Italian nurses and midwives. Nurs Health Sci.

[15] Middleton A, Emery SD, Turner GH (2010). Views, Knowledge, and Beliefs about Genetics and Genetic Counseling among Deaf People, Sign Language Studies.

[16] Binshihon SM, Alsulami MO, Alogaibi WM, Mohammedsaleh AH (2018). Knowledge and attitude toward hemoglobinopathies premarital screening program among unmarried population in western Saudi Arabia. Saudi Med J.

[17] Moussa S, Al-Zaylai F, Al-Shammari B, Al-Malaq KA (2018). Knowledge and attitude towards premarital screening and genetic counseling program among female university students, Hail region, Saudi Arabia. International Journal of Medical and Health Research.

[18] Altaany Z, Khabour OF, Al-Taani G (2019). Knowledge, Beliefs, and Attitudes Concerning Genetic Testing Among Young Jordanians. J Multidiscip Healthc.

[19] Olwi D, Merdad L, Ramadan E (2016). Knowledge of Genetics and Attitudes toward Genetic Testing among College Students in Saudi Arabia. Public Health Genomics.

[20] Middleton A, Hewison J, Mueller RF (1998). Attitudes of deaf adults toward genetic testing for hereditary deafness. Am J Hum Genet.

[21] Al-Farsi OA, Al-Farsi YM, Gupta I, Ouhtit A (2014). A study on knowledge, attitude, and practice towards premarital carrier screening among adults attending primary healthcare centers in a region in Oman. BMC Public Health.

[22] Ibrahim NK, Bashawri J, Al Bar H, Al Ahmadi J (2013). Premarital Screening and Genetic Counseling program: knowledge, attitude, and satisfaction of attendees of governmental outpatient clinics in Jeddah. J Infect Public Health.

[23] Alghamdi AM, Alqadheb AF, Alzahrani AM, Aldhahri AS, Alsharif ZM (2016). Knowledge of premarital screening among male university students in Riyadh, Saudi Arabia. Int J Med SciPublic Health.

[24] Zaien SZ, El Sayed HA, Ibrahim HA, Elgzar WT (2021). Empowering deaf and hard hearing females toward premarital counseling and genetic screening: An educational intervention based on empowerment model. Afr J Reprod Health.

[25] Bener A, Al-Mulla M, Clarke A (2019). Premarital Screening and Genetic Counseling Program: Studies from an Endogamous Population. Int J Appl Basic Med Res.

